# Perceptions of psychosocial disability amongst psychiatric service users and caregivers in South Africa

**DOI:** 10.4102/ajod.v3i1.146

**Published:** 2014-12-12

**Authors:** Carrie Brooke-Sumner, Crick Lund, Inge Petersen

**Affiliations:** 1 School of Applied Human Sciences, Discipline of Psychology, University of KwaZulu-Natal, South Africa; 2 Alan J. Flisher Centre for Public Mental Health Department of Psychiatry and Mental Health, University of Cape Town, South Africa

## Abstract

**Background:**

In many parts of South Africa there is little support for people with psychosocial disability caused by schizophrenia, beyond provision of psychotropic medications. Appropriate community-based psychosocial rehabilitation interventions are a crucial element of mental health service development.

**Objectives:**

This study aimed to use an explanatory model of illness framework to document experiences of illness, disability and recovery amongst service users with schizophrenia and their caregivers in a poorly resourced area in the North West Province. Data were used to provide recommendations for a contextually appropriate non-specialist facilitated group psychosocial rehabilitation intervention.

**Method:**

Eighteen in-depth individual interviews were conducted: nine with schizophrenia service users and nine with caregivers. Interviews were conducted by two trained field researchers; both clinical psychologists fluent in the first language of participants. All interviews were recorded, translated and transcribed. Data were thematically analysed using NVivo 9.

**Results:**

Participants linked the illness to witchcraft, poverty and stress. Family conflict was recognised in the course of the illness, causing stress and challenges for emotional well-being. Knowledge of diagnosis and biomedical treatment was minimal. Key factors recognised by service users as promoting recovery were the ability to work, and the support of traditional healers and religious structures.

**Conclusion:**

Based on the findings of this study, a group psychosocial rehabilitation intervention emerged as a recommendation, with the incorporation of psycho-education, adherence support, coping skills, and opportunities for income generation and productive activity. The importance of also enlisting the support of religious leaders and traditional healers in supporting recovery is emphasised.

## Introduction

Schizophrenia can be a chronic, highly disabling mental illness. Its associated disability can significantly undermine a person’s ability to function in their social environment. Schizophrenia was given the highest disability rating of all disorders studied in the 2010 Global Burden of Disease study (Whiteford *et al*. 2013). In the absence of nationally representative epidemiological studies, annual schizophrenia prevalence has been estimated at 1% in one South African province (Western Cape) (Kleintjes *et al*. 2006).

Psychosocial rehabilitation (PSR) helps people with schizophrenia on the journey of recovery by helping them gain skills and access resources that improve their capacity to live fulfilling and productive lives (Anthony & Farkas 2009). In many parts of South Africa there is, however, little support beyond provision of psychotropic medications, which are relatively widely available within primary health services (Lund *et al*. 2010).

There is a robust evidence base for psychosocial rehabilitation interventions promoting recovery in high income countries (HIC) (e.g. intensive case management (Dieterich *et al*. 2010), psycho-education (Xia, Merinder & Belgamwar 2011), interventions with families (Pharoah *et al*. 2010), life skills training (Tungpunkom, Maawan & Soares-Weiser 2012), cognitive behavioural therapy (Jones *et al*. 2011), cognitive rehabilitation (McGrath & Hayes Robyn, 2000). Growing evidence in low and middle-income countries (LMIC) indicates effectiveness of local adaptations of some of these interventions (e.g. Balaji *et al*., 2012; Chatterjee *et al*. 2011; Gutierrez-Maldonado, Caqueo-Urizar & Ferrer-Garcia 2009; Malakouti *et al*. 2009; Ran *et al*. 2003). Evidence for group-based psychosocial approaches, which may be more feasible than interventions delivered to individuals, is also growing in LMIC, for example group-based psycho-education in China (Weidong & Guoquan 2010; Zhang *et al*. 1998) and the Czech republic (Motlova *et al*. 2004); group-based social cognition training in Turkey (Tas *et al*. 2012); group-based cognitive rehabilitation in Malaysia (Alwi, Delisi & Nasrallah 2010) and Iran (Ali beigi *et al*. 2011) and group-based family intervention in Brazil (Cabral & Chaves 2010). In South Africa, recent studies have shown the potential for assertive community treatment (nurse- or social worker-led) (Botha *et al*. 2010; Botha *et al*. 2014) and group-based family therapy (nurse-led) (Asmal *et al*. 2013).

Insufficient resource allocation for psychosocial community-based services for recovery is well recognised in South Africa (Coetzee & Kemp 1982; Lund *et al*. 2010; Petersen & Lund 2011). Given the paucity of specialist mental health human resources (0.4 psychologists per 100 000 population; 0.1 social workers and occupational therapists per 100 000 population in the North West Province (the site of this study) (Lund *et al*. 2010), adopting a task sharing approach is indicated in line with global recommendations (Mari *et al*. 2009), the recent World Health Organization Mental Health Action Plan (WHO 2013) and South Africa’s National Mental Health Policy Framework and Strategic Plan (2013–2020) (Department of Health 2013).

## Objectives

The bio-psychosocial model of disability underlines the effects of mental illness on multiple linked ‘domains’ of an individual’s life (body, mind, and social interaction/participation) (King, Lloyd & Meehan 2007). The starting point for intervention is therefore an understanding of the lived experience of schizophrenia and psychosocial disability. This study aimed to document experiences and perceptions of illness, disability and recovery amongst service users with schizophrenia and caregivers, with the aim of informing a contextually appropriate community-based psychosocial intervention that could be facilitated by non-specialists within a resource-constrained setting.

### Contribution to the field

Understanding explanatory models of illness held by psychiatric service users with schizophrenia attending primary health care clinics for symptom management is important to inform the development of a socio-culturally acceptable psychosocial rehabilitation intervention at this level of care that can aid recovery within similar scarce resource contexts in South Africa.

## Research method and design

### Setting

This study is part of the Programme for Improving Mental Health Care (PRIME), a research consortium aiming to generate evidence for the implementation of programmes for priority mental disorders in low-resource settings (Lund *et al*. 2012). In South Africa, PRIME is a collaboration between mental health researchers and the South African Department of Health (DOH) in Dr Kenneth Kaunda District in the North West Province.

### Procedure

#### Sampling strategy

Service users were recruited using a convenience sampling approach: (1) through clinic registers from two primary care clinics (5 service users, 5 caregivers), and (2) through the North West Mental Health Society (4 service users, 4 caregivers). The North West Mental Health Society is affiliated to the South African Mental Health Federation (SAMHF) – a non-governmental organisation (NGO) providing mental health services and administering disability grants. Criteria for inclusion in the study were a confirmed diagnosis of schizophrenia or being a caregiver of a person with a schizophrenia diagnosis, and being over the age of 18. Service users interviewed were people whose symptoms were well managed and who accessed repeat medication from the clinics. The two interviewers were clinical psychologists who used their clinical judgement to establish whether participants were able to give informed consent and participate in the interview. Similarly, clinical judgement was used to assess the suitability of caregivers, and through this process one caregiver was referred to a psychologist for treatment of her depression.

#### Data collection

Eighteen semi-structured individual qualitative interviews were conducted, nine with service users and nine with their respective caregivers. The interview schedules were based on Kleinman’s concept of explanatory models of illness (EMI) (Kleinman 1980), and covered understanding of causes, experiences of symptoms, course (including experiences of stigma and discrimination), treatment (including interaction with service providers and experiences with disability grants), and healing or recovery. The interview schedules also included questions on the acceptability of a facilitated group approach. Interview schedules were reviewed and simplified to take into consideration possible lower levels of functioning of interview participants. Interviews were conducted by two clinical psychologists, both first-language Setswana speakers and fluent English speakers. One of the interviewers was also training to become a traditional healer, giving her a relevant perspective on issues relating to mental health in the community in question. The interviewers contributed to the development of the interview schedule to ensure consideration of cultural nuances. Interviews were conducted in service users’ first language (mainly Setswana, one English interview). All interviews were recorded, and then translated where necessary into English and transcribed.

#### Analyses

NVivo9 qualitative data analysis software was used to store data and conduct analysis. All interviews were coded by the first author. Framework analysis (Ritchie 1994) was used involving the following steps: Firstly, the transcripts were read and re-read to familiarise and immerse the researcher in the data. Secondly, a thematic framework corresponding to the interview schedules was generated for sorting data. Thirdly, the transcripts were coded, using the thematic framework developed, with additional themes being added as consecutive transcripts were analysed and new themes emerged. Further review and recoding of transcripts was carried out until no additional themes or subthemes emerged.

#### Ethical considerations

Permission was granted for this study from the University of KwaZulu-Natal and the University of Cape Town as part of the PRIME ethical approval (UKZN HSS/0623/012D; UCT HREC 412/2011). Approval for PRIME research was obtained from the provincial Department of Health. Potential participants were informed that there was no direct benefit to them of participating in the interview, other than the small grocery store voucher (R30) incentive. Participants were advised of the voluntary nature of their participation and of their right to withdraw from the study at any point. All participants provided written informed consent and permission to report findings, following an explanation of the research in their first language. The interviews were conducted in a private room and all personal identifying information was removed from the data. Hard copies of interview transcripts were stored in a locked office, and soft copies were stored on password locked computers.

## Results

Demographic characteristics of service users are given in Table 1. Demographic information on caregivers was not collected; however, all caregivers were female family members. Seven main themes emerged from the data.

**TABLE 1:** Demographic characteristics of service users interviewed.

Demographic	Service users (*N* = 9)	%
**Gender**		
Male	5	55
Female	4	45
**Age**		
18–20	0	-
21-30	1	11
31–40	2	22
41–59	6	67
**Education**		
No schooling	2	22
Primary education	3	33
Secondary and Post-Secondary	4	45
**Marital status**		
Not married	5	56
Married	1	11
Cohabiting	3	33
**Employment status**		
Employed	1	11
Unemployed	7	78
Student	1	11
**Housing**		
House	7	78
Rented room	2	22
**Number living in household**		
Under 16 years		
1-3 people	4	45
Over 16 years†		
1 person	0	-
2–3 people	6	67
4–6 people	2	22
**Sources of household income**		
Grants only	3	33
Grants and ad hoc work	1	11
Grants and salary	5	56

† Data missing for one participant.

### Understanding of causes and symptoms

#### Subtheme 1: Understanding of causes

Participants commonly had more than one understanding of the cause of their illness and impairments. Witchcraft was most commonly believed to be the cause: ‘A person might get naked and walk around the streets undressed due to her mental state that has changed after being bewitched’ (Service user participant 5).

One service user made the link between their illness and the stress caused by living in extreme poverty:

‘People should have money and should be given jobs because if you do not have money, you become stress[*ed*]. This stress exacerbates your illness. When you have money you don’t become mentally ill.’ (Service user participant 2)

Two service users identified conflict in their family as a cause of their illness. Three others attributed their illness to an experience of violence; a traumatic brain injury in childhood and smoking cannabis. Three caregivers attributed the illness to witchcraft, with one explaining that it could be due to a ‘calling’ to become a traditional healer. Other caregivers had different explanations – one linked the illness to their family member’s experience of being in prison, two believed that it was a result of pregnancy, and two attributed the worsening of the illness to the seasons.

#### Subtheme 2: Experience of symptoms and behaviour

When talking about their experience of the illness, service users focused on the physical impact and caregivers focused on behavioural aspects. Service users reported the disturbing nature of hallucinations, predominantly auditory and visual:

‘… I saw witches doing their private things. They were jumping over flames of fire. I saw them frying meat and jumping over the fire.’ (Service user participant 8)

They also reported unpleasant physical symptoms (some of which could be medication side effects), including feeling ‘heavy and confused’, dizziness, ‘feeling strained at the back of my head’, feeling a lack of control over their body, lack of concentration, difficulty sleeping, and disturbing dreams. One service user described how her inability to concentrate affected her ability to function:

‘I take a piece of paper [*list of things to buy*] each time I go to the shops. Even though I always have a piece of paper with me, I always wonder what to buy … and I feel so dizzy.’ (Service user participant 8)

The majority of caregivers related their experience of the illness to their relative’s disorganised, violent or destructive behaviour (e.g. burning property, beating children, walking the streets aimlessly, refusing to bath):

‘We get scared and sometimes when she fights she can really hurt someone because she [*is*] very strong.’ (Caregiver participant 14)

### Perceptions on the course and treatment of Illness

#### Subtheme 1: Knowledge of diagnosis

Knowledge of diagnosis was low amongst service users and caregivers. None were able to give the diagnosis that the service user had received in their clinic records. Three service users believed that their illness could be cured completely and one did not believe that he was ill. Two service users and three caregivers specifically mentioned that when they went to health services they were given medication, but that the illness was not explained to them:

‘They haven’t told me what kind [*of illness*] it is. She [*service provider*] says there are different mental illnesses, but they haven’t told me which type he has.’ (Caregiver participant 12)

#### Subtheme 2: Experiences of pharmacological treatment

Overall knowledge on medication and side effects was low. None of the service users talked about their medication by name. The majority (eight) service users reported good medication adherence and an appreciation of its benefits. However, five also noted challenges to adherence, including being unable to get to the clinic to collect medication, forgetting an appointment, or forgetting to take medication due to symptoms. Five service users said that a family member helped them to be adherent. This is in line with caregiver reports that they helped ensure that the service user they cared for took their medication. By contrast, several caregivers described verbal and physical fighting over medication:

‘He tells me to take them [*pills*] myself and see what they do to me’. (Caregiver participant 17)

Three service users expressed frustration linked to the different medications prescribed:

‘They never give me the right medication, one day they give me these pills and the next time I come they give me different ones. When they give me different medication, I have headaches and pain in my body’. (Service user participant 7)

#### Subtheme 3: Impact on emotional well-being

Service users and caregivers described how the illness had a significant negative impact on their emotional well-being. Two service users (both female) described how their illness caused them high levels of anxiety.

‘Sometimes I feel like I’m running without knowing where I’m running to, as if something was chasing me.’ (Service user participant 8)

Three service users mentioned having a short temper and one described his frustration at his inability to do things independently.

‘I feel I’m inconveniencing the person who helps me if I’m unable to complete a certain task … I’m supposed to complete it on my own’. (Service user participant 5)

Other emotional difficulties service users described included the loneliness of stays in hospital, and social isolation felt in everyday life. Caregivers described feelings of hurt and sadness caused by the service user’s behaviour towards them (three caregivers), fears of the service user’s violent or aggressive behaviour (three caregivers), embarrassment at their behaviour (e.g. lack of hygiene, two caregivers) and fears for their safety (e.g. when walking on the road, three caregivers). Six caregivers expressed a sense of loss in terms of how their lives could have been, had their family member not had this illness:

‘She [*service user*] could have been very successful, she used to love singing gospel and that’s all that she ever wanted to do. I could also have been successful.’ (Caregiver participant 14)

#### Subtheme 4: Family conflict as an impact of the illness

Family conflict was described by both service users and caregivers. Two service users (both female) felt the illness had a negative impact on their intimate relationships. Two service users said their family believed that they acted deliberately to cause difficulty for the family. One service user then noted how this lack of understanding caused a worsening of his symptoms:

‘They [*family members*] say “You behave as if you are crazy and you are not.” When they say that, the voices attack me and become louder.’ (Service user participant 2)

One caregiver noted how the illness had caused ‘chaos’ for the whole family, and particularly had hurt the children of the service user:

‘Yes, we are always stressed … at night and in the morning you will find that we discipline him for all his mistakes. Then during the day … we shout at him.’ (Caregiver participant 17)

### Conceptualisation of and support for recovery

When talking about recovery and their ideas of a positive future, responses on recovery concepts identified from international literature (e.g. meaning and purpose) were limited. Several service users focused on meeting basic needs (e.g. food, clothing), indicative of the challenging conditions in which they live.

#### Subtheme 1: Healthy relationships

Three service users expressed their need to have positive relationships with those around them, including family members and service providers:

‘I need to stop shouting at them [*nurses, doctors, social workers*] and start to be polite with them. And with my family so that there are not conflicts.’ (Service user participant 2)

#### Subtheme 2: Importance of productive activity and work

The majority of service users highlighted the importance of productive activity in their lives, including household chores and gardening:

‘When I’m feeling better, I’m able to do things for myself such as washing dishes and clothes. But when I’m unwell I can’t do anything so my children help me.’ (Service user participant 7)

Seven service users highlighted the importance of work in their perception of a positive future:

Interviewer: ‘When do you feel like your life has meaning and purpose?’

Respondent: ‘When I feel like what I was supposed to do for the day is completed … just like the job I used to do … I was doing it with all my heart.’ (Service user participant 2)

Two service users noted that it was their lack of skills and education that was a challenge to finding employment. Three service users said their illness prevented them from working, noting the effect of symptoms and impairment in preventing them from performing work-related tasks.

Four caregivers believed the service user they cared for was unable to work due to being unable to do physically intense work, being aggressive, feeling tired, or due to their destructive behaviour and the unwillingness of employers to hire those with mental illness:

‘She burns people’s things, no, she can’t work. Others have hired her before, thinking that she was normal, but as time goes on they see that she is not well.’ (Caregiver participant 16)

#### Subtheme 5: Support of traditional healers

Five service users said they had or would consult a traditional healer, either of their own accord, or at the direction of family. Three service users believed that the traditional healer had helped them understand or improve their symptoms. Two service users felt that they had not been effective, and three caregivers emphasised that they did not view traditional healers as effective:

‘Modern doctors don’t understand muti [*traditional medicine*] that people give you in your food. I think a traditional healer would help me understand the causes of this illness better.’ (Service user participant 7)

Three service users and one caregiver indicated that they thought both Western and traditional approaches could aid in their recovery:

‘His [*traditional healer*] name is Mr. Peter, he helped and the pills helped me too. When I left Witrand [*psychiatric hospital*] I was feeling much better and I went to the traditional healer who helped me to recover fully.’ (Service user participant 6)

#### Subtheme 6: Significance of religion

Service users and caregivers indicated that organised (Christian) religion and faith in God was key to coping with their disability and life circumstances. Several participants indicated that instead of worrying they put themselves ‘in the hands of God’:

‘I still see the man who attacked me … but I’ve never opened a case against him, I just give everything to God.’ (Service user participant 8)

Caregivers gained comfort from their belief that God would protect the person they care for, and give them strength to fulfil their caring role:

‘Each time the illness starts he just leave[*s*] the house and we do not know where he’s staying. God is the one who will protect him.’ (Caregiver participant 10)

### Experiences of stigma and discrimination

#### Subtheme 1: Experiences of health services

Three service users described good treatment at hospitals and clinics. Conversely, three service users described being treated badly by nurses. One service user described an experience of stigma related to his receiving a disability grant:

‘They [*nurses*] said … when we buy clothes we spend their money because it comes from their taxes. They said I should give them money because I get a grant and I’m arrogant.’ (Service user participant 3)

#### Subtheme 2: Treatment by family

Only one service user reported being treated with dignity and respect by family members. Seven service users reported various forms of ill treatment by family members, including verbal abuse, being refused food, being ignored when in need of help, and being prevented from leaving the home. Two caregivers provided evidence of verbal and physical abuse by other family members:

‘There was a time when I went to my uncle to ask for help and he just shut the door in my face. I had been attacked with a knife, I only wanted help.’ (Service user participant 8)

#### Subtheme 3: Experiences in the social environment

One service user said he was generally well treated and respected in his community, and four caregivers also described good treatment by neighbours. However four service users described various forms of ill treatment including being called ‘crazy’ or ‘lunatic’, being accused of committing crimes, being refused help or financial loans, being refused service at shops, and being ridiculed.

‘They just accuse me of things that I don’t know about. They say that I kill people and they want to [*take*] revenge … they even call me a rapist.’ (Service user participant 5)

Community members also took advantage of service users’ impairments. This included engaging the service user in work and not paying them, short-changing them at shops and sending them on unpaid errands. Two caregivers believed their female family members had been sexually abused.

### Experiences associated with disability grants

All service users interviewed were receiving disability grants from the South African Social Security Agency (SASSA) on the basis of their psychosocial disability. Two caregivers said that the grant was extremely helpful to their families, enabling them to eat even though no one was working. Grants were not always effectively managed, with three caregivers having to use grant money to pay off debts that the service user had incurred through buying meat, clothing or alcohol. Four service users reported managing the money from their grant themselves, and four said their caregiver managed the money. Three of these reported that their caregivers took advantage by spending money on themselves or withholding money. Conversely, one caregiver felt misunderstood due to being accused of mismanagement of the grant:

‘She says that the community is saying that I spend her money on my own things … and that hurts me because I know that I’m not misusing her money.’ (Caregiver participant 16)

Despite this regular income, one service user articulated his desire to work and how the fear of losing his grant discouraged this.

‘I wish I could go back to the shop and start working again. But they said that if I continue working then I won’t get my pension money.’ (Service user participant 5)

### Caregiver burden

Significant burden emerged as a defining characteristic of the experience of caregivers. Several articulated a strong sense of responsibility to their family member with schizophrenia, exacerbated by the lack of involvement of other family members:

‘Whenever she [*gets*] ill, everyone in the house backs off and the responsibility is left to me.’ (Caregiver participant 14)

One caregiver had suicidal thoughts related to her inability to cope with life as a caregiver.

### Acceptability of facilitated group approach

Four service users said they would be happy with a group facilitated by non-specialists. Two of these service users however highlighted the importance of these non-specialists being trained in the specifics of their mental illness. One service user said he would not join because he went to the hospital regularly and would get advice from the doctor. He did acknowledge that a reason for him to join would be to share his knowledge and experience with others. One other service user said they would not join without giving a reason. The remaining three service users did not respond to this question, seemingly due to lack of understanding of the facilitated group format. Seven caregivers said they would join groups; however, one said she did not have the time and one said the family member she cared for would not be able to participate due to difficulty with communicating.

Motivations for service users joining a group included doing something ‘on the side’ to increase income, learning new skills, sharing experiences with people in a similar situation, keeping busy, avoiding substance abuse, being reminded to take medication, and having positive social interactions:

‘I would go to talk about my problems so that we can help each other.’ (Service user participant 8)

Caregivers’ motivations included gaining support and comforting one another, giving and gaining advice on how to cope with their caring role, sharing experiences and motivating each other, finding a release for their stress and emotions, and building friendships:

‘If you talked to them [people in the group] about living with a mentally challenged patient, then they could empathise with you. They would also share how they handled similar situations.’ (Caregiver participant 17)

## Discussion

Participants in this study held little knowledge on biomedical aspects of the illness (symptoms, treatment) in line with previous research in the North West Province, which indicated low levels of knowledge of schizophrenia amongst service providers (Modiba *et al*. 2001). Traditional beliefs on causation were also common, in line with previous studies showing that attitudes, beliefs and experiences relating to schizophrenia are substantially different in South Africa from Western conceptualisations (Mbanga *et al*. 2002; Mosotho, Louw & Calitz 2011). Service users’ conceptualisation of their recovery from the illness was characterised by high value placed on engagement in productive activities and the ability to work as reported previously in South Africa by service users and user advocates (Kleintjes, Lund & Swartz 2012; Van Niekerk 2009;). Linked to the importance of work, the fact that some service users described their inability to meet their basic needs for survival (e.g. food) indicates the pressing unmet need for a broad spectrum of supportive services. Despite the support net provided by disability grants, evidence from this study of the hardships for service users and their families caused by living in poverty, similar to those documented for service users with schizophrenia in other parts of South Africa (Swartz *et al*. 2006), reaffirms the crucial need for psychosocial rehabilitation interventions to promote recovery and reintegration of service users into their community and into productive activity, including employment (Coetzee & Kemp, 1982; Lund *et al*. 2010; Petersen & Lund 2011). Support from traditional healers was important for recovery, in line with the South African context in which traditional healers provide significant psychosocial support for other conditions (Campbell-Hall *et al*. 2010). Religion or spirituality has been suggested to provide problem-solving strategies, a source of social support, the derivation of meaning, and improved self-worth for those with severe mental illness (Shah *et al*. 2011), and the reporting of the significance of religion for recovery and coping by service users and caregivers suggests this is the case in this context.

The study confirmed significant social disability, social isolation and stigma and discrimination experienced by service users reflective of the South African context in which schizophrenia is stigmatised significantly more than other mental disorders (Sorsdahl & Stein 2010). Caregivers in this study also shouldered a heavy burden, and both service users and caregivers were negatively affected by family conflict and lack of coping skills for dealing with the impact of the illness on their emotional well-being and the functioning of their families.

### Recommendations for psychosocial rehabilitation intervention

Data from this study provide direction regarding the design of a facilitated group psychosocial rehabilitation intervention as detailed in Table 2.

**TABLE 2:** Recommended elements of a contextually appropriate group psychosocial intervention for service users with schizophrenia and caregivers in the North West Province, South Africa.

Type of intervention	Need identified	Recommended strategy for addressing need
Service user intervention	a. Orientation of facilitators to experiences and perspectives of service users	Training on the impact of poverty, stigma and discrimination and stress on service users. Highlight importance of traditional and religious explanatory models of illness (EMI) and related coping strategies. Highlight role of traditional healers and religious organisations in providing psychosocial support but emphasise adherence to medication.
	b. Psycho-education on causes, symptoms, course	Dedicate significant time in meetings for psycho-education adapted from evidence-based approaches. Increase insight into individual nature of recovery and address expectations regarding cure. Increase insight into coping strategies for symptoms. Emphasise self-management and involvement in care.
	c. Medication literacy and adherence support	Dedicate time in meetings for sharing accurate information on medication. Involve a nurse or specialist if possible. Address different types of medication, side effects and the fact that medication may change based on availability.
	d. Reduce stress associated with family conflict and improve emotional well-being	Provide information on role of family conflict in stress and relapse. Skills building for managing anger and stress and dealing with conflict, coping skills for stress and anxiety. Improve money management skills and communication around disability grants.
	e. Tools for dealing with social isolation, experiences of stigma and discrimination	Skills building on problem management and healthy thinking. Prioritise time for sharing of experiences on stigma/discrimination in meetings, encouraging peer support Encourage group members to support each other outside of facilitated meetings. Organise social activities in addition to group meetings.
	f. Productive activities to help to reduce poverty and stress and improve role functioning	Include information on importance of productive activity for recovery. Link to skills development and employment opportunities. Enable service users to contribute more effectively to household tasks.
Caregiver intervention	g. Orientation of facilitators to experiences of caregivers	Training on impact of stress, burden, poverty, missed opportunities, lack of hope for future.
	h. Psycho-education on causes, symptoms, treatment, course, role of conflict	Increase empathy for service users, reduce fear.
	i. Improve caregiver ability to create an environment supportive to recovery	Share strategies for coping with conflict. Ensure opportunities for sharing of experiences and coping strategies.
	j. Reduce caregiver burden, improve emotional well-being	Share information on accessing resources in community, caring for the caregiver. Encourage environment of peer support Incorporate sharing of information with extended family. Build coping skills for dealing with stress and anxiety Encourage participation of male caregivers.

#### Psycho-education

Low levels of awareness amongst service users and caregivers of the diagnostic condition (even though awareness of the presence of a mental illness was common) highlight a need for the provision of clear and accurate information. Lack of knowledge limits service users’ ability to participate in their care (Kleintjes, Lund & Swartz 2013a), a key aspect of recovery. The South African primary health care system is currently piloting a system that emphasises integrated chronic disease management, based on the Chronic Care Model (CCM) (Wagner *et al*., 2005) This emphasises, inter alia, the need for informed and motivated service users who are able to take charge of managing their condition (Battersby *et al*. 2010 – Table 2b). Thus, the proposed facilitated group intervention is timely and aligns with the development of the South African health system.

#### Adherence support

Low levels of knowledge on medication caused confusion for service users and difficulties with adherence. Similarly, service users had low levels of knowledge of medication side effects and some perceived possible side effects as symptoms of their illness (e.g. cognitive difficulties). Medication literacy for service users (and caregivers who have a key role in promoting adherence) is a clear need. In the South African context, non-adherence may also be linked to services users’ EMI (Bhagwanjee *et al*. 2008) and lack of understanding of these on the part of service providers grounded in biomedical approaches. To effectively support adherence, facilitators of the proposed group intervention will need to encourage health-promoting behaviours (e.g. adherence, knowing when to get help) alongside a traditional explanatory framework (Table 2; section a, h). This aligns with earlier work in South Africa indicating that service users with mental illness maintain traditional beliefs on the causation of their illness, whilst still seeking and valuing biomedical treatment for alleviating symptoms (Lund 1998). The current study also reinforces the need to move beyond the stereotype of the direct conflict between ‘Traditional African’ and ‘modern Western’ understandings of mental illness and recovery (Lund 1998).

#### Coping skills for family conflict and emotional well-being

Although some schizophrenia service users in South Africa see themselves as being valued and respected by caregivers despite the lack of community-based supportive services (Manamela *et al*. 2003), in this study family conflict was evident as a source of stress for service users and caregivers. Their contrasting responses in some areas of this study suggest potential reasons for conflict, for example caregivers reported fighting over medication, whereas service users reported significant caregiver support for adherence; service users expressed a desire to be productive, whereas caregivers believed they were unable to do this. Clearly the experience of illness for service users and caregivers is exacerbated by living in poverty, and similar to findings of a recent study of family therapy for schizophrenia in South Africa (Asmal *et al*. 2013), the proposed intervention will benefit from adaptation of conflict management strategies to specifically address poverty and its associated challenges.

Stress and anxiety were important challenges to the emotional well-being of service users and caregivers in this study, and these too may be related to the presence of conflict in the family. Beyond psycho-education on schizophrenia, there is a need for increasing mental health literacy on other mental health problems, and building resilience and coping through sharing skills and practical strategies for managing stress and anxiety (Table 2; section d, k).

#### Coping skills for stigma and discrimination

Data from the current study indicate that stigma and discrimination constitute a significant barrier to social integration of service users. Internalised stigma associated with this illness may further compound the burden of psychosocial disability. This aligns with earlier work in the North West Province showing schizophrenia service users to have particular needs in the areas of improving social networks and community integration (Modiba *et al*. 2001). Increasing knowledge and empathy amongst caregivers (Table 2; section i) may work towards reducing stigma and discrimination. It is equally important to equip service users with coping strategies for dealing positively with experiences of external stigma and discrimination (unlikely to change in the short-term – Table 2; section e), as well as internalised/self-stigma. Finding effective ways for increasing awareness and understanding of mental illness in the wider community are clearly crucial but beyond the specific scope of the proposed intervention. However, there has been a call for greater prominence of service users in awareness raising and other advocacy initiatives in South Africa (Kleintjes *et al*. 2006), and facilitated groups may be an effective way to help mobilise service users towards advocacy and the development of policy and services in which they currently have little participation (Kleintjes *et al*. 2010).

#### Income generation and productive activity

Although disability grants provide a safety net from absolute poverty for service users and their families, data from this study also indicate they can be a source of conflict. Improving money management and communication skills is indicated (Table 2; section d). Many service users in this study emphasised that social integration in the form of working and earning an income would be the cornerstone of recovery. In the wider context of poverty, unemployment, and multiple dependents on disability grants, a significant motivator for participation in groups may be opportunities for income generation to add to income derived from grants (Table 2; section f), as has been the case in user-led groups in other African countries which have incorporated strategies for sustainable livelihoods such as group savings schemes and income-generating cooperatives (Kleintjes, Lund & Swartz 2013b). Data from this study also indicate the importance of productive activity in the household other than work and income generation. Improving service users’ contribution to household tasks may help to boost self-esteem, with the additional benefit of helping to reduce burden on caregivers (Table 2; section f).

#### Addressing caregiver burden

The frequency of reporting in this study of significant caregiver burden suggests a need for a separate intervention to support family/caregivers. Families who are best equipped to care for a person with schizophrenia are educated on the illness and engaged in seeking help (Rose 1996). Access to information on the supportive resources available in the community acts as a buffer to the burden of care (Caqueo-Urizar & Gutierrez-Maldonado 2006). The proposed intervention should therefore incorporate these aspects, as well as enabling caregivers themselves to share their own coping strategies with each other (Table 2; section k). In this study, all caregivers interviewed were female, corresponding to the global picture of caregiving (Esplen 2009). Low levels of perceived support from other family members have been associated with higher caregiver burden (Biegel *et al*. 1994) and for countries such as South Africa, where the social welfare system (e.g. sheltered living) is inadequate, it may be beneficial to focus on ways of strengthening extended family relations (Ohaeri 2001) and increasing the role of men in caregiving (Table 2; section k).

#### Promoting acceptability

The facilitated group approach seems to be acceptable to service users and caregivers in this study – only two people said they would not join if accessible groups were set up. There was an expressed need for group facilitators to have specific training on schizophrenia and a particular sensitivity to the impacts psychosocial disability has on service users’ lives. This highlights the need for specifically designed training for group facilitators. This training should also incorporate orientation towards the acceptance of service users’ EMI. In this study, service users and caregivers linked their experiences of illness or disability to stress and poverty, and empathy for this reality will be key to promoting acceptance. Religion as a coping strategy was commonly reported in this study. Making provision for understanding and supporting religion and spirituality (Ali beigi *et al*. 2011) rather than assuming this is a result of psychopathology (Reiland & Lauterbach 2008) may further increase the acceptability of the proposed intervention (See Table 2; section a).

Both caregivers and service users in this study indicated that mutual support and sharing experiences would be an important motivator for participating in groups. This grounding in peer support as a facilitator of recovery has been the basis of user-led organisations that have developed elsewhere in Africa (Kleintjes *et al*. 2013b). While a user-led group approach is likely to be the most acceptable form of intervention, service users in this study were disempowered by their lack of knowledge on their illness, lack of education, and their lack of access to basic services. A facilitated group approach may be a positive first step in moving towards service users’ self-organisation and advocacy. The groups would also benefit from linking with the South African Mental Health Advocacy Movement to develop over time in line with this model of peer-led groups.

## Limitations

This study was limited in having a relatively small sample (comprised of two subgroups, with differing views and experiences) and in following a convenience sampling strategy. This may have introduced homogeneity in the life circumstances and experiences of participants. A response bias may also have been introduced due to the setting of interviews in a health facility. The study was limited by difficulties in gaining responses to all interview questions due to the level of functioning of some service users. The clinical training of the interviewers and their understanding of the symptomatology of schizophrenia was a strategy employed to ensure the best data possible were gained. However, this could also have introduced a limitation in terms of reducing their perspective and sensitivity to salient cultural issues relevant to participants understanding of mental illness and recovery. Similarly, the one interviewer’s position as a traditional healer may also have introduced a bias towards this perspective. Both interviewers received specific training on the interview schedule aiming to reduce these possible sources of bias. Other limitations include the potential for researcher biases in this type of qualitative research analysis.

## Conclusion

This study has highlighted the multiple challenges faced by service users with schizophrenia and their caregivers in South Africa, encompassing not only the experience of disability, but also the impact of high levels of poverty. The study has identified potential elements of a contextually and culturally appropriate group psychosocial intervention, linked to the perceptions and experiences of service users with schizophrenia and their caregivers in the Dr Kenneth Kaunda District, North West Province.
